# Modelling 3D craniofacial growth trajectories for population comparison and classification illustrated using sex-differences

**DOI:** 10.1038/s41598-018-22752-5

**Published:** 2018-03-19

**Authors:** Harold S. Matthews, Anthony J. Penington, Rita Hardiman, Yi Fan, John G. Clement, Nicola M. Kilpatrick, Peter D. Claes

**Affiliations:** 1 Murdoch Children’s Research Institute, Melbourne, Australia; 2 Royal Children’s Hospital, Melbourne, Australia; 3 Department of Paediatrics, University of Melbourne, Melbourne, Australia; 4 Melbourne Dental School, University of Melbourne, Melbourne, Australia; 5 Cranfield University, Cranfield, UK; 6 Processing Speech and Images, Department of Electrical Engineering, Katholieke Universiteit, Leuven, Belgium; 7 Medical Imaging Research Centre, Universitair Ziekenhuis, Leuven, Belgium

## Abstract

Many disorders present with characteristic abnormalities of the craniofacial complex. Precise descriptions of how and when these abnormalities emerge and change during childhood and adolescence can inform our understanding of their underlying pathology and facilitate diagnosis from craniofacial shape. In this paper we develop a framework for analysing how anatomical differences between populations emerge and change over time, and for binary group classification that adapts to the age of each participant. As a proxy for a disease-control comparison we use a database of 3D photographs of normally developing boys and girls to examine emerging sex-differences. Essentially we define 3D craniofacial ‘growth curves’ for each sex. Differences in the forehead, upper lip, chin and nose emerge primarily from different growth rates between the groups, whereas differences in the buccal region involve different growth directions. Differences in the forehead, buccal region and chin are evident before puberty, challenging the view that sex differences result from pubertal hormone levels. Classification accuracy was best for older children. This paper represents a significant methodological advance for the study of facial differences between growing populations and comprehensively describes developing craniofacial sex differences.

## Introduction

Many disorders present with characteristic abnormalities in craniofacial shape, emerging through abnormal pre- or postnatal growth. Precise descriptions of how these abnormalities develop and change can inform our understanding of their underlying biology and facilitate diagnosis. To this end, we present a framework for characterising population-level differences in 3D craniofacial growth trajectories and classification from craniofacial shape. As a proxy for a disease-control comparison we illustrate the method by characterising sex differences as they emerge through childhood and adolescence. Note, we use the term "growth" loosely to refer to changes in shape that are associated with changes in age in a cross-sectional sample.

Traditionally, craniofacial measurement has relied on simple distances and angles between anatomical landmarks^1^, which give only a limited representation of the surface under study. 3D photography and advances in image analysis have now achieved rapid, automatic measurement of the entire outer surface of the craniofacial soft tissue^2–4^. This allows the anatomy to be quantified as a dense cloud of point co-ordinates providing a high resolution description of the surface. Averaging these corresponding points within a disease group and within a control group to produce ‘prototypical’ faces provides a spatially dense description of the differences between the two groups^5–10^. These representations have also been used to learn classification models for diagnosis from craniofacial shape^5,8^. However, these studies are limited in that they have amalgamated data from mixed ages to create a single pair of prototypes or a single classification model. This neither accounts for the fact that the differences between the groups may change with age, nor provides any insight into the different growth patterns of the two groups.

In general, systematic differences in anatomy result from systematic differences in pre- or postnatal growth. Growth may differ in terms of growth rate (one group grows faster than the other), growth direction (the growth vectors of the two groups point in different directions) or in some combination of the two. Importantly, the nature of these differences in growth can’t be inferred directly from the differences in shape; the same differences in shape can arise in different ways. For example a difference in nose width could arise from both population’s noses widening at different rates, or alternatively from one population’s nose narrowing and the other’s widening. Different growth patterns may imply quite different biology and should be investigated where possible. Previous studies have used the first principal component (PC) of shape variation to describe growth direction. The simple linear regression coefficient of scores on the first PC, regressed onto age, has been used to measure growth rate and to infer that slow growth rate explains part of the facial phenotypes in Wolf-Hirschhorn and Williams Syndromes^9,11^. While providing valuable insights, these descriptions are limited in that they assume consistent rate and direction of growth (i.e., they are linear), when both rate and direction may vary with age. Furthermore the first PC is defined without any reference to age and may, therefore, not correspond to growth. Hutton *et al*.^12^ used kernel regression^13,14^ to define non-linear growth trajectories explicitly from age but did not address directly how to estimate growth rate and direction from them.

We present a method of describing facial differences as they change throughout childhood and adolescence and for classification at different ages, using an extension of Hutton *et al*.’s kernel regression. The approach estimates the expected head shape, growth rate and direction at each age, in each group to describe how the facial differences emerge. We demonstrate the method by examining when and how sex differences in craniofacial shape (sexual dimorphism) emerge, based on a large cross-sectional sample of children and adolescents. Sexual dimorphism is of considerable interest to anthropologists and anatomists. The combination of state-of-the-art methodology and a large sample size spread over childhood and adolescence makes this, to the best of our knowledge, the most comprehensive analysis of the emergence of sexual dimorphism to date.

## Method

### Ethical approval

This work was granted ethical approval by the human research ethics committee of the Royal Children’s Hospital (RCH), Melbourne (#29008I) and was carried out in accordance with the approved protocol. Informed consent was obtained from all participants (if aged over 18 years) or their legal guardian (if aged less than 18 years).

### Sample

**Figure 1:**
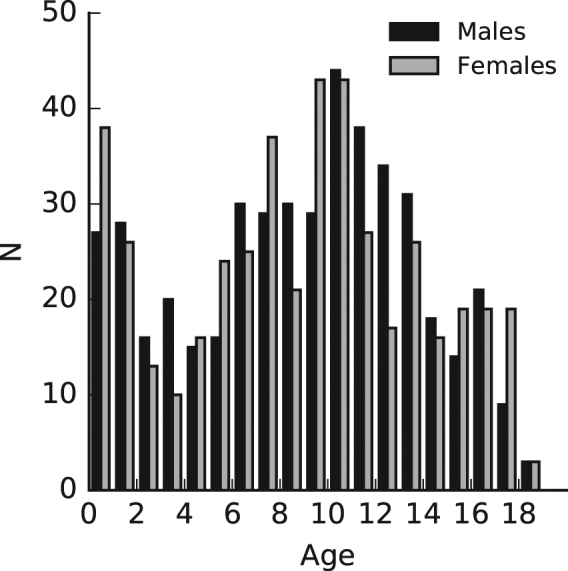
Counts of boys and girls at each age.

The sample consisted of 452 boys and 442 girls (range: 0.05-18.60 years old). Participants were included if the parent, when asked on the consent form, did not report that the child had a disorder that was likely to affect craniofacial growth. We assume parents would have reported any disorder requiring major craniofacial surgery. However, problems requiring more minor interventions, like braces, may not have been reported. These may be common among the adolescents in this study. Participants were only included if their self-reported ethnicity was Australian, European or North American. Frequency counts for each age are shown in Fig. 1.

Participants were recruited through flyers distributed around the RCH and by advertisement on the RCH’s Facebook page. Participants were often visitors to patients or patients for issues not affecting craniofacial growth. Participants were also recruited during visits to primary and secondary schools in the greater Melbourne area.

### Image acquisition

A 3D photograph of the entire head, including the face, ears and neurocranium, was taken of each participant using either the 3dMD 7-pod or mobile 5-pod system (http://www.3dmd.com/). Images were taken either at the RCH or at nearby primary and secondary schools. A tight-fitting stocking, placed over each participant’s neurocranium pressed the hair tightly, and as evenly as possible, to the scalp, allowing the camera system to record it. This is the best method available for controlling the effect of hair on the image, however the actual imaged shape of the neurocranium will still partly reflect the thickness and shape of the compressed hair. Findings on the neurocranium should be treated with some caution.

### Image measurement

The 3D surface in each image was sampled at 28,861 points, by gradually warping a generic template head into the shape of each image^2,3^. The point co-ordinates of this warped head then represent the shape of head in the 3D photograph. Some younger children had their mouths partially open, and these were corrected using the approach described by Matthews *et al*.^15^. We also recorded the physical size of each head as the mean distance of all points on the head from their centroid. All heads were made symmetrical by creating the mean of the original image and its reflected copy, so as to focus our analysis on the symmetric component of shape variation^16^. All heads were scaled to unit centroid size and their rotation and location were standardised using generalised Procrustes analysis (GPA) with a robust Procrustes transformation^17^.

### Modelling growth trajectories

**Figure 2:**
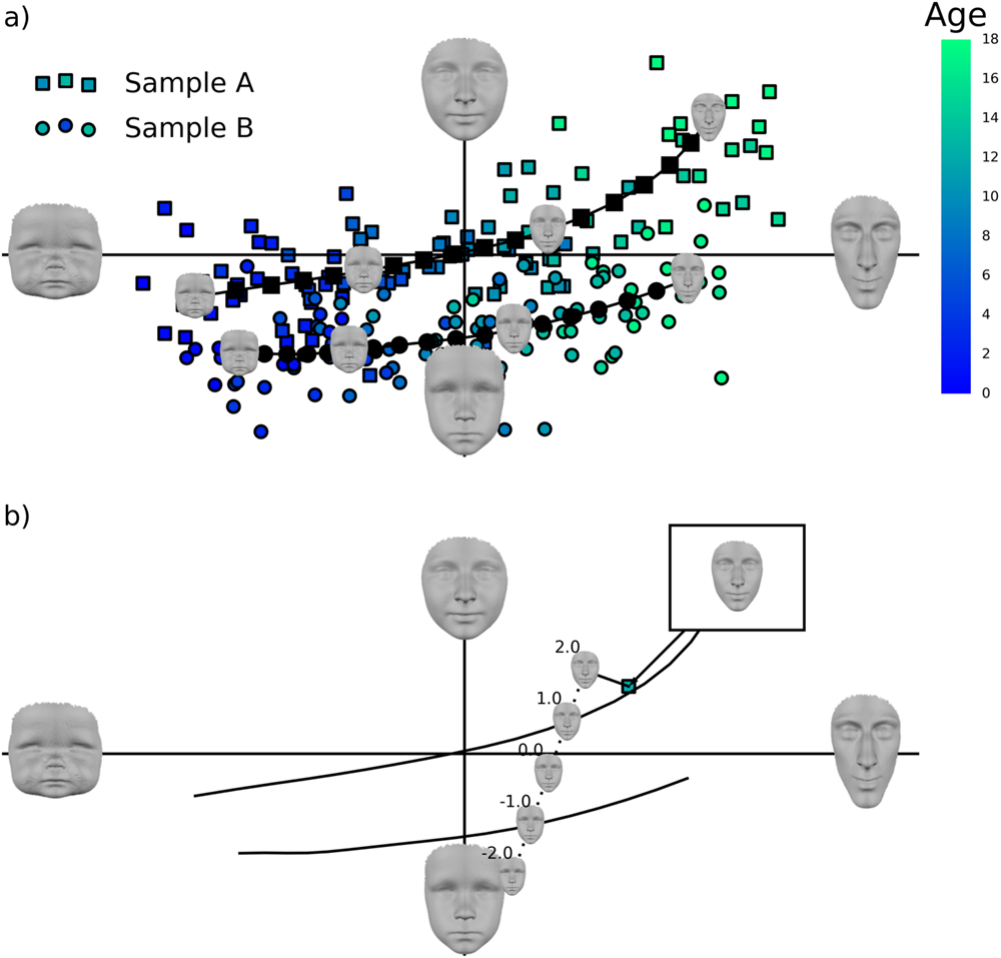
Kernel regression and classification in shape space. In both figures the axes are the first and second principal components, which are two orthogonal directions through the shape space that explain the most and second-most variance. The aspects of facial variability the axes represent are illustrated by the large grey faces. The data points show simulated data. In a) each individual (indicated by the markers) can be thought of as a location in the space that codes their shape. Kernel regression (illustrated by the curved lines) chart a curve through this space that describes how shape changes as a function of age (indicated by marker colour). Locations on these lines correspond to expected ‘typical’ heads for each age, some of which are superimposed onto the line. b) illustrates calculating the score for classification. This is equivalent to interpolating heads between and beyond the two age appropriate expected heads (illustrated by the heads on the dotted line), then finding the one most similar to the test case.

Further details of the implementation and mathematics are reported in Supplementary Text S1. Briefly, after alignment by GPA, a point-configuration can be interpreted as a location in a shape or face space where each axis of the space corresponds to a point co-ordinate. Such a space is shown schematically in Fig. 2.

Modelling growth trajectories is like charting a curve through this shape space (see Fig. 2 and Supplementary Movie S1). We model growth trajectories of shape and size for boys and girls using four separate kernel regressions. Kernel regression^13,14^ models the non-linear relationship between predictor (in this case age) and response variable(s), without assuming that the relationship follows any particular mathematical function. To describe the expected value (expected head shape or size) of either group at any target age, *a*, we use a ‘kernel’ of observations from the group that are most similar in age to *a*. The kernel does not have hard boundaries, but rather the influence of each observation is weighted so as to smoothly decline with increasing distance from the target age. The expected value for each age was predicted using a weighted linear partial least-squares regression model of the response variable(s) onto age^18,19^. This regression model also estimates growth rate and growth direction at *a*. In the regression of shape, it is a vector of regression coefficients for all point-coordinates and describes the predicted change in the head at that age, essentially estimating a 3D growth vector at each point on the head. The width of the kernel was tuned to avoid over-and under-fitting using a repeated grid search (see ref.^20^ and Supplementary Text S1). The regressions of size used a kernel width of 0.75 years, the regressions of shape used a kernel width of 2.75 years. Cases within one kernel width of *a* have the most influence, cases more than two kernel widths from *a* have close to zero influence.

We summarise the overall magnitude of sexual dimorphism in shape using the Procrustes distance^21^ between expected heads at each age. Its significance is determined against null distributions calculated by permuting the group (sex) labels, re-fitting the regressions with the same kernel width, and recomputing the Procrustes distances 10 000 times. The overall growth rate within each group at each age was measured as the mean length of the estimated growth vectors, over all points on the head, at that age. We visualise the differences between expected heads and growth vectors at each point on the head for each age using colour-coded heads.

As the proportion of cases either below or above the target age approaches zero (such as at the edges of the sampled ages), the local and overall kernel regression becomes only an extrapolation. Therefore, although our sample ranges from 0.05 years to 18.60 years, we only evaluate the model between 1.12 and sixteen years old. These limits were defined so that the ratio of the sum of weights of cases older than *a* to the sum of weights of cases younger than *a*, or its inverse, never drops below 0.4. This threshold was chosen by trial and error so as to exclude results that were clearly artefactual. For example, beyond age sixteen we observed an increase in estimated growth rate in both groups, which is biologically implausible.

### Classification from shape

In order to classify each participant as either ‘boy’ or ‘girl’ we produce a score that reflects how masculine or feminine their head is, where the definition of masculine-feminine adapts to their age. For each participant, we take their age (*a*), evaluate the expected heads for that age on both growth trajectories and calculate the participant’s ‘projection’ onto the direction between the expected heads. This is illustrated in Fig. 2 and is equivalent to interpolating heads in between and beyond the two expected heads and then finding the most similar one to the participant. This score is then normalised according to half of the Procrustes distance between the expected heads. The result is that a score of minus one means that the observation is most similar to the female expected head and a score of positive one means it is most similar to the male expected head. Values beyond one and minus one indicate the head is more masculine or feminine than average respectively. Values close to zero indicate an androgynous head.

We assessed the performance of this classifier using repeated k-fold cross-validation. For 100 repetitions we split the sample into ten ‘folds’. These folds were constructed so that the composition of males and females of each age was proportional to the composition of the whole sample. For each head in each of the ten folds we computed their score as above, defining the expected faces using the remaining nine folds. The results were then binned into four age brackets (less than five, five to ten, ten to fifteen and over fifteen), to assess if the classifier performs differently for different age brackets. Classifier performance for each fold was measured by the area under the receiver operator characteristic curve (AUC) and the correct classification rate, using a threshold of zero. Means and confidence intervals of these values were calculated over all 1000 folds. To visualise the overlap between the groups we produced histograms of participant’s mean scores over all 100 repetitions.

An open-source implementation of the automatic image measurement algorithm is available at https://github.com/TheWebMonks/meshmonk. All subsequent analyses were implemented in Python by the lead author. 3D visualisations were produced using Mayavi (http://docs.enthought.com/mayavi/mayavi/).

### Data availability

As images of heads are potentially identifiable and children are a vulnerable population, images were collected without consent for broad data sharing. Therefore the raw images cannot be made publicly available online. Requests for access can be directed to AP (tony.penington@rch.org.au), and will be subject to approval by the project steering committee and the RCH ethics committee.

Each participant’s head shape, coded non-identifiably as principal component projections, head size, necessary participant metadata and Python code for the analyses are available at https://github.com/harrymatthews50/Modelling_Craniofacial_Growth_Trajectories.

## Results

### Overall size and shape differences

**Figure 3:**
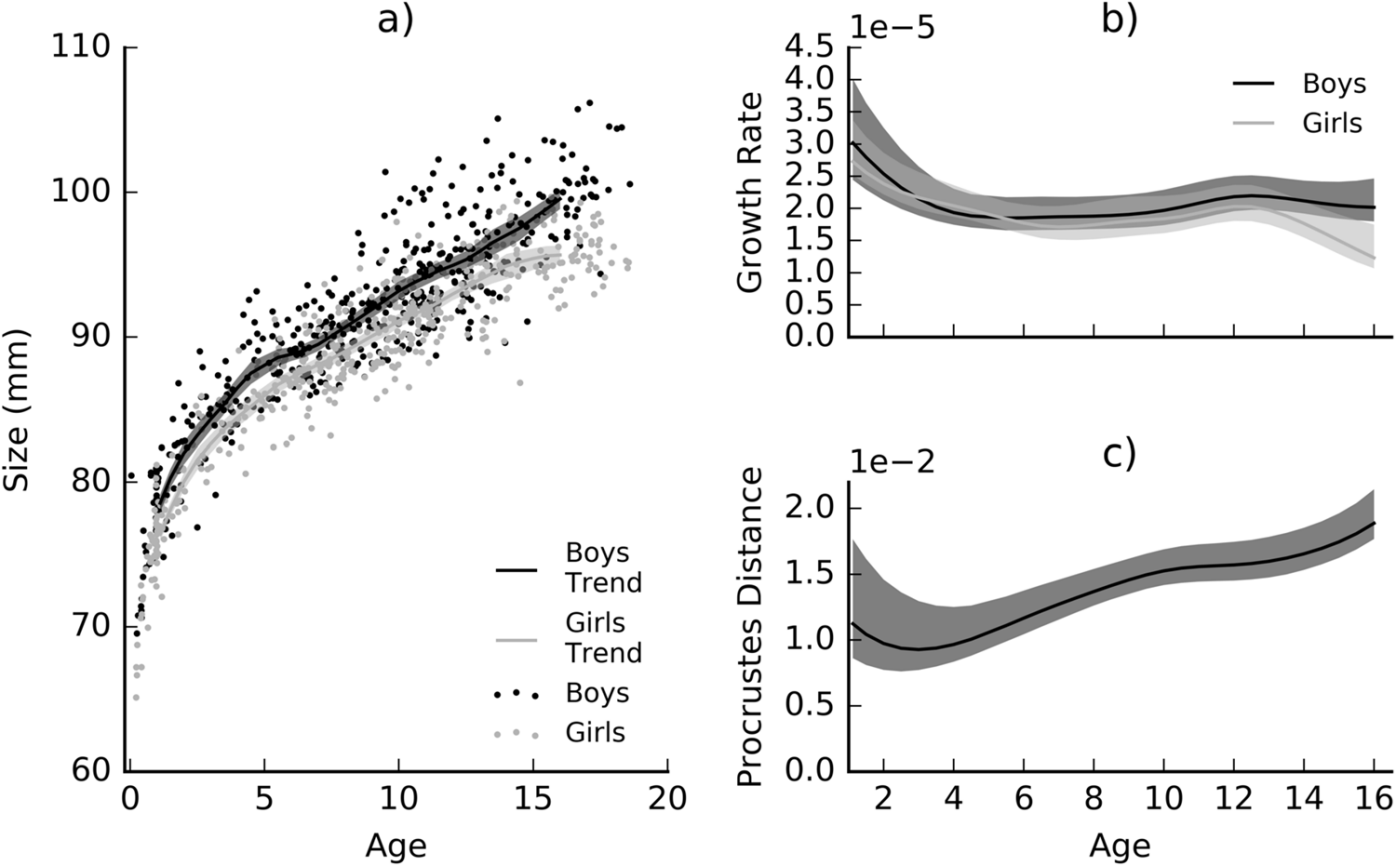
Overall trends in growth and sexual dimorphism. a) describes the change in size of boys’ and girls’ heads. Size is calculated as the mean distance of each point on the head from the centroid of all points in mm. Lines are the kernel regression trend-lines for each group. b) describes the change in the size of sexual dimorphism (Procrustes distance). c) compares the rate of change in shape between males and females. In all plots the filled regions indicate the 95% confidence intervals of the estimate. These were calculated by resampling with replacement and recomputing the estimates 10 000 times.

Figure 3a plots overall head size as a function of age. In general heads of girls are smaller than the heads of boys. The size of this difference is fairly constant and both groups change at a similar rate. This is up until age fourteen when the size of girls’ heads plateaus but the size of boys’ heads continues to increase, exaggerating the difference between the groups. Figure 3b compares growth rate between boys and girls. Similarly to size, their growth rates are similar up until about age fourteen, where the change in girls’ heads slows down, but does not in boys’. The peaks in growth rate during adolescence are not different between the sexes, as might be expected from differences in pubertal timing. However, when the analysis is repeated including size and shape together (by omitting the scaling operation from the GPA) there are different peaks in growth rate, with girls’ growth accelerating from approximately age eleven and boys’ accelerating from approximately age twelve (Supplementary Figure S1).

Figure 3c shows the overall magnitude of shape sexual dimorphism as a function of age. The size of this difference was significant at all ages (*p* < 0.05). Exact p-values are reported in Supplementary Table S1. The magnitude of dimorphism declines up until about age three, then it increases in two phases between ages five and ten and between ages twelve and sixteen. The magnitude of dimorphism possibly continues to increase beyond age sixteen.

### Dimorphic features

**Figure 4:**
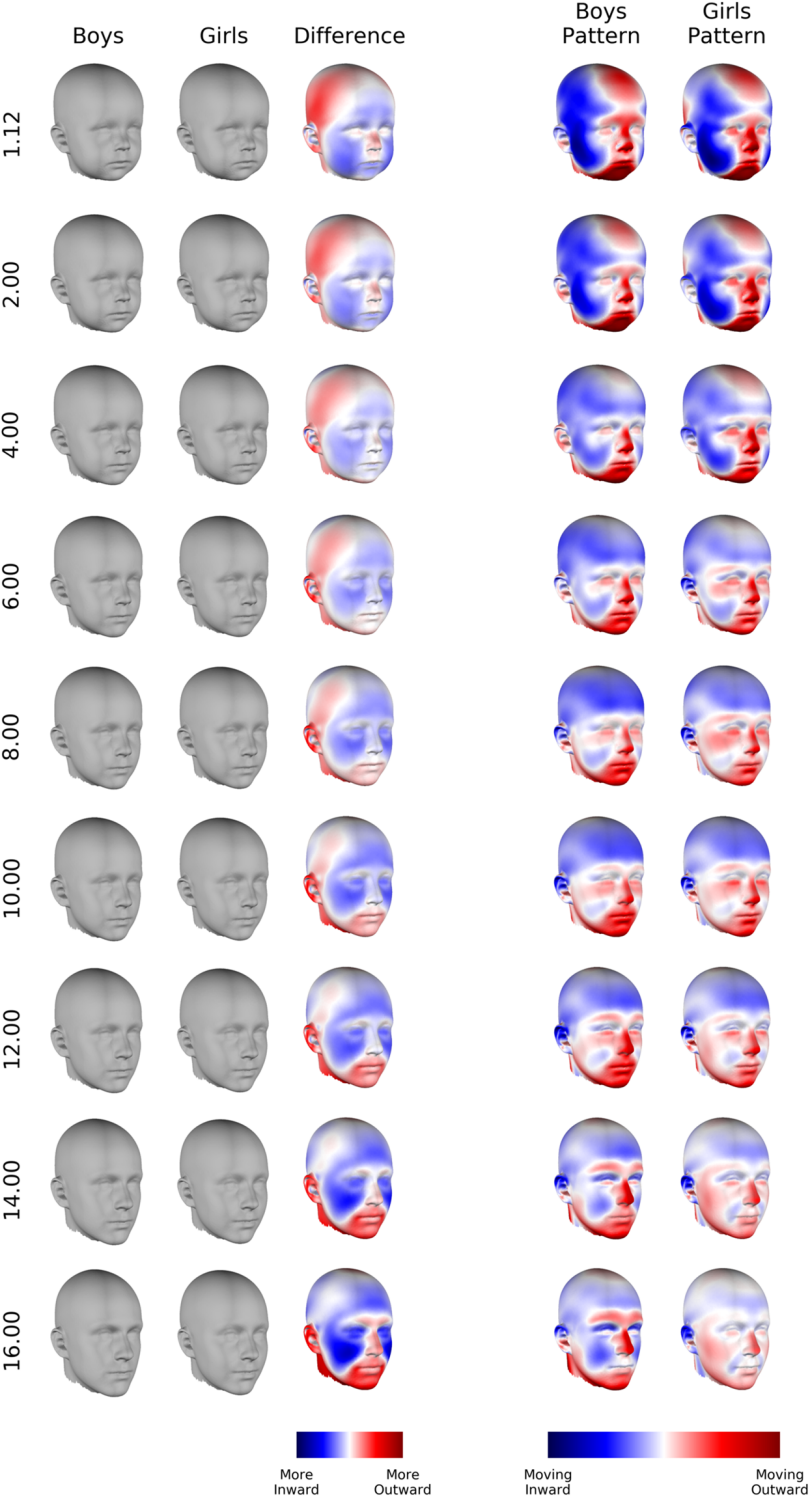
Growth patterns and comparison of expected faces. The first three columns describe sexual dimorphism. The grey heads are the expected images. ‘Shape difference’ indicates how the expected images are different in the inward/outward direction. Blue indicates points are more inwards on the boys’ images than the girls’ images. Red indicates the points are more outwards. The last two columns illustrate the growth patterns of boys and girls. These indicate the amount of change occurring in the inward/outward direction at each point. Stronger colours indicate more change.

The first three columns of Fig. 4 illustrate the difference between girls and boys at some example ages. The first two show the expected heads for boys and girls. The third (‘Shape Difference’) shows the difference between them in the direction of the girls’ surface normals (locally inward/outward). Expected heads and colour-maps for a much finer sampling of ages are compiled into an animation of the heads ‘growing up’ (Supplementary Movie S2). Difference in the lateral (left/right), vertical (superior/inferior) and depth (anterior/posterior) directions are available in Supplementary Figures S2–S4 (all ‘Shape Difference’ maps here and in the supplement use the same colour scale, so that they are comparable). ‘Morphs’ that exaggerate the difference between boys and girls are in Supplementary Figure S5.

The primary features that distinguish the two groups at sixteen years old are that in boys relative to girls: 1) the forehead slopes posteriorly, 2) the brow-ridge protrudes anteriorly, 3) the chin is positioned anteriorly and inferiorly, 4) the upper lip is positioned anteriorly and inferiorly, 5) the nose protrudes anteriorly and 6) the buccal region is flatter.

### Differences in growth rate and direction in the emergence of dimorphice features

**Figure 5:**
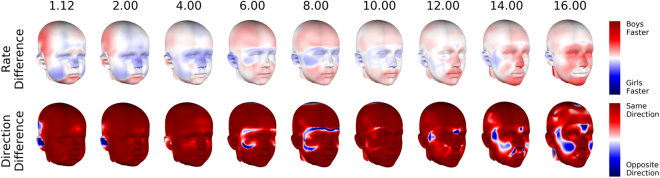
Comparison of growth patterns between boys and girls. ‘Rate Difference’ compares the growth rate of males and females at each point on the head. Red indicates males are changing faster, blue indicates females are changing faster. ‘Direction Difference’ compares the growth directions at each point on the head. Red indicates the growth vectors are pointing in the same direction, blue indicates they are pointing in the opposite direction.

In general these differences in shape must result from differences between the growth patterns of the two groups. The growth patterns for each group are plotted in the final two columns of Fig. 4. These indicate the predicted rate of change at each point in the inward/outward direction. Change along the lateral, vertical and depth axes is also available in Supplementary Figures S2–S4. Figure 5 (top row) compares the rate of change between boys and girls. All growth patterns and rate comparisons use the same colour scale. Figure 5 (bottom row) compares the direction of change between the two groups and is coloured according to the angle between growth vectors of each group at the same point on the head. These results are condensed into descriptions of how each dimorphic feature emerges below:

#### Forehead slopes posteriorly

The expected boys’ forehead is retro-positioned relative to girls’ at the earliest time (1.12 years). In younger children both foreheads protrude forward, but the boys’ protrude less (see Supplementary Figure S5). In general the foreheads in both groups become more retro-positioned as they get older, but the boys’ do so faster, until their forehead actually slopes posteriorly.

#### Brow ridge protrudes anteriorly

At ages twelve and fourteen the brow ridge of both groups become more prominent but the boys’ do so faster, giving rise to differences in the brow ridge which are evident by age sixteen.

#### Chin is positioned anteriorly and inferiorly

At most ages the boys’ chin grows faster than girls’ and there is no obvious difference in the direction of growth vectors of the two groups on this region. These vectors have a strong anterior (Supplementary Figure S3) and inferior (Supplementary Figure S4) component. Thus the chins of both groups project anteriorly and inferiorly, but boys do so faster. This results in a difference in chin position by age eight.

#### Upper lip is positioned anteriorly and inferiorly

From age six the upper lip differences emerge like the chin differences. That is, by a difference in rate of change along anterior and inferior growth vectors. The difference is evident from age twelve.

#### Nose protrudes anteriorly

This difference is present up until age two and then disappears. The boys’ nose starts to change more rapidly than the girls’ at about age twelve, leading to this difference re-emerging by age fourteen.

#### Buccal region flatter

While this difference is present at all ages it becomes more exaggerated between ages six and ten mostly due to a difference in growth rate (although on a small portion of the cheek there are also differences in growth direction). Points on the cheeks are displaced inwards for both groups, but boys are displaced faster. Between ages twelve to sixteen this difference continues to become exaggerated, but due to different growth direction of this region at this time. The points on the girls’ cheeks move outward while those on the boys’ cheeks move inward.

#### Additional features

At age 1.12 the anterior and posterior (not visible in the printed figure) neurocranium are compressed inwards while the lateral part projects more outwards (more laterally). This indicates a more brachycephalic head shape in boys than girls. Over time, the lateral portion of the neurocranium is displaced inwards (medially). This occurs faster for males than females, resulting in a reduction of the lateral projection of the neurocranium. This difference is totally absent by sixteen.

### Classification from shape

**Figure 6:**
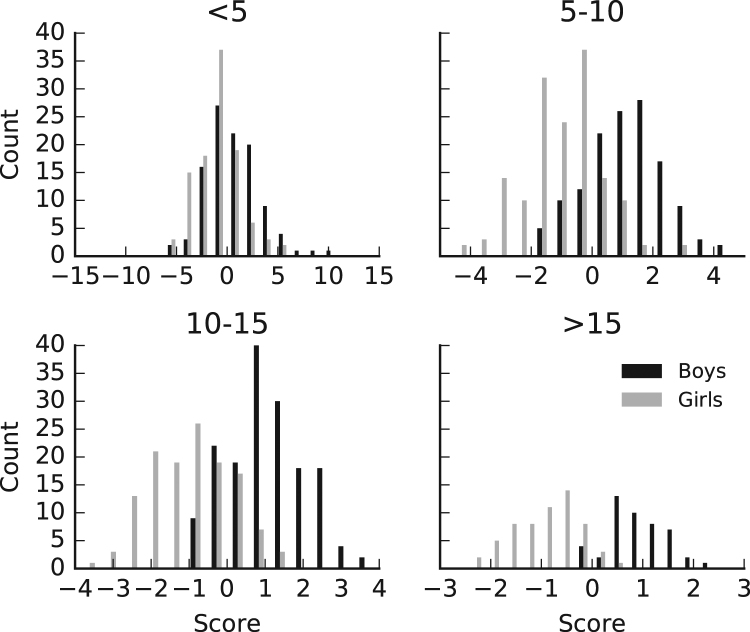
Distributions of scores for the classification analysis for males and females.

Table 1 shows statistics of classifier performance. AUC of 0.5 indicates chance performance. Classification was not significantly better than chance for participants less than five years old but was in the 5–10 and 10–15 age brackets. Performance was near perfect in those over 15. Figure 6 plots the distributions of scores for each age bracket. The groups overlap almost completely in the bracket 0–5 and become more separated in the older brackets.

**Table 1:** Classifier performance statistics. AUC is the area under the receiver operator characteristic curve. AUC of 0.5 indicates chance performance. Values outside the brackets are the mean value over the 1000 folds. Values in brackets are the 95% confidence intervals.

	AUC	Boys % Correct	Girls % Correct
<5	0.65 (0.40,0.88)	54.81 (25.00,87.50)	70.16 (41.67,100.00)
5–10	0.87 (0.73,0.98)	80.19 (57.14,100.00)	80.03 (58.78,100.00)
10–15	0.91 (0.80,0.99)	81.79 (62.50,100.00)	78.91 (58.30,100.00)
15–20	0.98 (0.88,1.00)	91.59 (60.00,100.00)	93.81 (66.67,100.00)

## Discussion

Differences in head shape between groups are most frequently analysed by comparing simple measurements, or single average faces. However, understanding how these differences emerge and change can provide insights into the underlying biological and genetic mechanisms. In this study we introduce a framework for describing and analysing the emergence of shape differences between populations and apply the method to the question of how differences in craniofacial shape emerge between males and females. The combination of a large sample size, covering most of childhood and adolescence, and spatially-dense analysis is unique in the literature on craniofacial sexual dimorphism. We first discuss our results in this context, followed by considering the broader applications of the methodology and its relationship to existing approaches.

At sixteen we observe a protruding brow ridge and nose; a chin and upper lip that are positioned more anteriorly and inferiorly; and a flatter buccal region in boys relative to girls. These features have been consistently observed in adults^16,22,23^. Many soft-tissue studies exclude most of the forehead, focussing only on the facial shell but, in those that include it, it is generally found to be sloping backwards more in adult males than females^24,25^. Given the proximity of frontal bone to the skin, findings on this area can be directly related to the hard tissue anatomy. Consistent with our results, hard tissue studies have observed increased volume, surface area and base area of the brow ridge^26,27^ and an increased forehead inclination^28^ in adult male skulls, relative to females’. In our analysis, the brow ridge prominence develops independently of the superior portion of the forehead, indicating growth by isolated deposition on this region; not by deposition at cranial sutures which would shift the whole frontal bone anteriorly. Increased soft tissue thickness on the upper lip and chin has been documented in adults^19,29^. Shape differences in these areas may represent differences in soft tissue depth, in combination with different positioning of the mandible and maxillary bones. The nose differs in both its cartilaginous parts and the bony nasal bridge. This is consistent with previous observations of skulls that revealed males have a more anterior projection of the most inferior point of the nasal bone and a larger anterior nasal aperture^30^. Differences in the buccal region probably mostly reflect differences in soft tissue, including the buccal fat pad and facial musculature, rather than the underlying hard-tissue.

With regard to how these differences emerge: the retro-positioning of the forehead is present at age 1.12 and becomes more exaggerated primarily due to differences in growth rate along similar growth vectors between boys and girls. Although differences in the nose are also present at age 1.12 these disappear between age two and four. At about age twelve the growth rate of the nose for boys becomes faster than for girls, giving rise to the differences we see at sixteen. Differences in the chin and upper-lip emerge from differences in growth rate by ages eight and twelve respectively. Differences in the buccal region are present at all time points, but become more exaggerated over time. This occurs first through differences in growth rate and then, around puberty, through differences in growth direction. Overall, we observe that there are two phases in the emergence of sexual dimorphism. During the first, between ages five and ten, the anterior and inferior positioning of the chin in boys appears and differences in the buccal region and forehead continue to become exaggerated. The second is from about twelve onwards. During this period differences in the nose, brow ridge and upper-lip emerge and differences in the chin, buccal region and forehead continue to become exaggerated. Accompanying the increased differences between the groups we observe decreasing overlap in the distributions of male-female scores and better classification accuracy.

Traditionally it has been assumed that sexual dimorphism emerges primarily at puberty, resulting from an influx of sex-hormones^31,32^. Contrary to this view, a few studies have reported sex-differences in facial measurements in pre-pubescent children^33–35^. Bulygina *et al*.^25^ and Kesterke *et al*.^34^ observed shape differences in children down to age 0.5 and three respectively. They were limited however in that they both used relatively sparse landmark configurations to represent the anatomy. Furthermore, Bulygina only analysed the shape described in 2d in a lateral cephalogram and only used a very small sample size. Previously our lab demonstrated retro-positioning of the forehead, flattening of the buccal region, a slightly more brachycephalic head shape and an enlarged nose in boys relative to girls at one year-old^15^. Our findings at age 1.12 replicate this study using a different sample. We also find that the differences in the buccal region and forehead are maintained and become more exaggerated, gradually developing into adult patterns of dimorphism, whereas the relative brachycephaly disappears entirely. The differences in the nose disappear, only to reappear during adolescence.

Our study adds to the growing body of literature demonstrating that sexual dimorphism is present very early in life. Our results show aspects of this early dimorphism continue to develop during the pre-pubescent period, suggesting that adult sexual dimorphism is not all attributable to sex hormones released at puberty. Neave *et al*.^36^ argued that the ratio of testosterone to estrogen experienced in utero affects the underlying organisation of craniofacial structures, and that these differences are then activated at puberty. This is corroborated by evidence that masculinity of facial shape in adult males is correlated with this hormone ratio^37,38^. The observed differences in the pre-pubescent period may be due to uterine hormone levels, activated prior to puberty, or they may represent sex-linked facial characteristics, not directly attributable to hormone levels.

The growth trajectory of a particular species or group may be adaptive, in an evolutionary sense, and be an object of natural selection^39^. This implies that differences between growth trajectories of males and females can inform our understanding of how natural selection, including sexual selection, has operated differently on the two groups. Although outside the scope of this paper we consider how our findings relate to three important anthropological hypotheses: 1) growth directions are the same and sexual dimorphism emerges by males developing faster over the same amount of time; 2) growth directions are the same and males and females develop at the same rate, but males do so for longer; 3) growth directions are not the same. In general, it is not possible to prove similarity of growth directions. However, our results indicate dimorphism of the nose, chin, forehead and brow-ridge are largely a product of different rates of change, not different directions. Differences in the buccal region result from differences in growth rate between six and ten and from different growth directions between twelve and sixteen.

This study represents an important methodological advance in the description of group level differences, and classification therefrom, in growing populations. Previous studies have used average faces based on samples of mixed ages, ignoring the fact that the differences may change over time^5–9^. Others have grouped data into age brackets and constructed mean heads for each age bracket^34^. Binning requires decisions about where to divide the data, which may affect the results. Furthermore, if the brackets are large, it is likely that changes occurring within the age bin will be missed. Hutton *et al*.^12^ used a kernel regression model to define an expected head at any age, as the weighted mean of a local kernel of ages. Since it can be evaluated for any age the expectation is effectively continuous, unlike discrete age bins. They used a weighting system that emphasises those cases closest in age to the target age, maximising how well the expected head represents the target age. In this work, we extend their approach by using a weighted regression model, instead of the weighted mean, to define the expected head for each age. The regression also predicts change in each point co-ordinate, estimating a 3D growth vector for each point on the head. This allows pointwise comparison of growth rate and growth direction, facilitating a deeper understanding of how the differences between the populations emerge. It is also more robust to uneven sampling than the weighted mean (see ref.^40^ and Supplementary Text S1).

We extend Hutton’s approach further and apply it to binary classification. Traditional classification models (including linear discriminant analysis, Euclidean distance to centroids and support vector machines) attempt to discriminate groups based on a set of variables. In a binary classification scenario they fit a single surface through the space of these variables (e.g. shape space) that best divides two groups. These models can be applied to new cases, which may make them valuable diagnostic aids^5,8,41,42^. Beyond simply diagnosing cases categorically, if the discriminating surface is a plane, the signed distance of an observation from it can be calculated easily and interpreted as a continuous measure of phenotype expression. This may identify subclinical cases of disorders that exhibit only a reduced form of the full phenotype. The classification method applied here produces a score describing where the case sits on the axis between age appropriate expected faces. This is related to traditional models in that this score is proportional to the signed distance from a discriminating plane orthogonal to this axis. In contrast to traditional approaches the location and orientation of this plane adapts to the age of the case being classified.

One important application may be in the study and assessment of foetal alcohol spectrum disorders (FASDs). FASDs are a spectrum of neurocognitive deficits observed in children exposed to alcohol in utero. The more severe manifestations are accompanied by specific facial anomalies including a shortened palpebral fissure, thin vermillion line, flat philtrum and reduced head circumference^43^. Recent studies have also observed subtle facial differences in alcohol exposed children not diagnosed with a FASD^6,44^. The classic facial differences diminish in adolescence^45^. The time-course of the more subtle aspects is unknown, but could be investigated with our methodology. Given the emerging evidence for a spectrum of facial effects, a shape based facial phenotype score, like the one described here, may flag potential cases for further assessment that might otherwise be undetected.

Although for the current analysis we draw on a very large database of images, such large samples are unlikely to be available for every study. The described approach compensates for small sample size by increasing the width of the kernel of ages used to estimate each expected head. In Supplementary Text S1 we describe how to optimise this width for any dataset. Increasing the width of the window linearizes the growth trajectory; as the size of the window gets very large the model approaches being a single partial least-squares regression. While this model, being linear, would clearly be a simplification of growth, even in this worst-case scenario, it still improves on the common method of using the first principal component. Specifically the first principal component is simply the dimension of greatest variation within the sample. It is also linear, but is constructed without any reference to age. Therefore there is no guarantee in principle that it relates to growth. Our approach, in contrast, always takes age into account in defining the growth trajectories.

An important limitation in modelling growth from age is that individuals mature at different rates. Age, therefore, only approximates maturation. A further limitation is that we did not adjust for allometry, the association between centroid size and shape^46^. The heads of boys are bigger than girls’ so the observed shape differences between them could reflect the effect of size on shape within the local age range, rather than the effect of sex. As centroid size is strongly correlated with age (data not shown), it is not appropriate to adjust for it in the current study. Doing so would essentially remove the effect of age on shape (growth), which is an important aspect of our analysis.

In this paper we describe an approach for analysing the emergence and change in craniofacial differences between two growing populations and for age-adaptive classification. We demonstrate the method by asking how the differences in head shape between boys and girls emerge. In doing so we comprehensively describe the emergence of craniofacial sexual dimorphism and add to the growing body of research indicating that there is sexual dimorphism long before puberty. Mostly the differences between the sexes emerge through boys growing at a faster rate than girls. In future the method can be used to chart changes in the distinctive facial appearances that characterise many disorders, including foetal alcohol spectrum disorders (FASDs). These changing descriptions can be used to better understand their underlying pathology and facilitate diagnosis from facial shape.

## Electronic supplementary material

Supplementary Information

Movie S1

Movie S2

## References

[1] Farkas, L. G. *Anthropometry of the Head and Face*. (Raven Press, 1994).

[2] Claes P, Walters M, Clement J (2012). Improved facial outcome assessment using a 3D anthropometric mask. Int. J. Oral Maxillofac. Surg..

[3] Snyders, J., Claes, P., Vandermeulen, D. & Suetens, P. Development and comparison of non-rigid surface registration and extensions. Report No. KUL/ESAT/PSI/1401, 1–55 (2014) at https://mirc.uzleuven.be/tools/download.php?root=MedicalImageComputing&UID=Ox1rVsXvR57nzedYDuypWw7G2QpeJQ.

[4] Hutton, T. J., Buxton, B. F. & Hammond, P. In *BMVC*. (eds R Harvey & A Bangham) 439-448 (Citeseer, 2003).

[5] Hammond P (2005). Discriminating power of localized three-dimensional facial morphology. Am. J. Hum. Genet..

[6] Suttie M (2013). Facial dysmorphism across the fetal alcohol spectrum. Pediatrics.

[7] Hammond P, Suttie M (2012). Large‐scale objective phenotyping of 3D facial morphology. Hum. Mutat..

[8] Cox-Brinkman J (2007). Three-dimensional face shape in Fabry disease. Eur. J. Hum. Genet..

[9] Hammond P (2012). Fine-grained facial phenotype–genotype analysis in Wolf–Hirschhorn syndrome. Eur. J. Hum. Genet..

[10] Shaweesh A, Clement J, Thomas C, Bankier A (2006). Construction and use of facial archetypes in anthropology and syndrome diagnosis. Forensic Sci. Int..

[11] Hammond P (2007). The use of 3D face shape modelling in dysmorphology. Arch. Dis. Child..

[12] Hutton TJ, Buxton BF, Hammond P, Potts HW (2003). Estimating average growth trajectories in shape-space using kernel smoothing. IEEE Trans. Med. Imaging.

[13] Nadaraya EA (1964). On estimating regression. Theory of Probability & Its Applications.

[14] Watson, G. S. Smooth regression analysis. *Sankhyā: The Indian Journal of Statistics*, *Series A*, 359–372 (1964).

[15] Matthews H (2016). Spatially dense morphometrics of craniofacial sexual dimorphism in one year-olds. J. Anat..

[16] Claes P (2012). Sexual dimorphism in multiple aspects of 3D facial symmetry and asymmetry defined by spatially dense geometric morphometrics. J. Anat..

[17] Claes, P. *et al*. Dysmorphometrics: The modelling of morphological abnormality. *Theoretical Biology and Medical Modelling* **9** (2012). 10.1186/1742-4682-9-5 PMC3297492 22309623

[18] Wold S, Ruhe A, Wold H, Dunn I (1984). WJ. The collinearity problem in linear regression. The partial least squares (PLS) approach to generalized inverses. SIAM J. Sci. Stat. Comp..

[19] Shrimpton S (2014). A spatially-dense regression study of facial form and tissue depth: Towards an interactive tool for craniofacial reconstruction. Forensic Sci. Int..

[20] Krstajic D, Buturovic LJ, Leahy DE, Thomas S (2014). Cross-validation pitfalls when selecting and assessing regression and classification models. J. Cheminform..

[21] Dryden, I. L. & Mardia, K. V. *Statistical shape analysis*. Vol. 4 (Wiley Chichester, 1998).

[22] Claes, P. *et al*. Modelling 3D facial shape from DNA. *PLoS Genet*. **10** (2014). 10.1371/journal.pgen.1004224 PMC3961191 24651127

[23] Hennessy RJ, McLearie S, Kinsella A, Waddington JL (2005). Facial surface analysis by 3D laser scanning and geometric morphometrics in relation to sexual dimorphism in cerebral–craniofacial morphogenesis and cognitive function. J. Anat..

[24] Velemínská J (2012). Surface facial modelling and allometry in relation to sexual dimorphism. HOMO.

[25] Bulygina E, Mitteroecker P, Aiello L (2006). Ontogeny of facial dimorphism and patterns of individual development within one human population. Am. J. Phys. Anthropol..

[26] Shearer BM, Sholts SB, Garvin HM, Wärmländer SK (2012). Sexual dimorphism in human browridge volume measured from 3D models of dry crania: a new digital morphometrics approach. Forensic Sci. Int..

[27] Garvin HM, Ruff CB (2012). Sexual dimorphism in skeletal browridge and chin morphologies determined using a new quantitative method. Am. J. Phys. Anthropol..

[28] Petaros A, Garvin HM, Sholts SB, Schlager S, Wärmländer SK (2017). Sexual dimorphism and regional variation in human frontal bone inclination measured via digital 3D models. Leg. Med..

[29] Manhein MH (2000). *In vivo* facial tissue depth measurements for children and adults. Journal of Forensic Science.

[30] Rosas A, Bastir M (2002). Thin-plate spline analysis of allometry and sexual dimorphism in the human craniofacial complex. Am. J. Phys. Anthropol..

[31] Enlow, D. H. & Hans, M. G. *Essentials of Facial Growth*. (WB Saunders Company, 1996).

[32] Bernstein RM (2017). Hormones and human and nonhuman primate growth. Horm. Res. Paediatr..

[33] Gaži-Čoklica V, Muretić Ž, Brčić R, Kern J, Miličić A (1997). Craniofacial parameters during growth from the deciduous to permanent dentition—a longitudinal study. Eur. J. Orthod..

[34] Kesterke MJ (2016). Using the 3D Facial Norms Database to investigate craniofacial sexual dimorphism in healthy children, adolescents, and adults. Biol. Sex Differ..

[35] Joffe TH (2005). Fetal and infant head circumference sexual dimorphism in primates. Am. J. Phys. Anthropol..

[36] Neave N, Laing S, Fink B, Manning JT (2003). Second to fourth digit ratio, testosterone and perceived male dominance. Proc. R. Soc. Lond. B. Biol. Sci..

[37] Schaefer K, Fink B, Mitteroecker P, Neave N, Bookstein FL (2005). Visualizing facial shape regression upon 2nd to 4th digit ratio and testosterone. Coll. Antropol..

[38] Weinberg SM, Parsons TE, Raffensperger ZD, Marazita ML (2015). Prenatal sex hormones, digit ratio, and face shape in adult males. Orthod. Craniofac. Res..

[39] Shea BT (1986). Ontogenetic approaches to sexual dimorphism in anthropoids. Hum. Evol..

[40] Hastie, T., Tibshirani, R. & Friedman, J. *The Elements of Statistical Learning*. (Springer, 2001).

[41] Hammond P (2004). 3D analysis of facial morphology. Am. J. Med. Genet. A.

[42] Weinberg SM (2008). Three-dimensional morphometric analysis of craniofacial shape in the unaffected relatives of individuals with nonsyndromic orofacial clefts: a possible marker for genetic susceptibility. Am. J. Med. Genet. A.

[43] Hoyme, H. E. *et al*. Updated clinical guidelines for diagnosing fetal alcohol spectrum disorders. *Pediatrics* **138** (2016). 10.1542/peds.2015-4256 PMC4960726 27464676

[44] Muggli E (2017). Association between prenatal alcohol exposure and craniofacial shape of children at 12 months of age. JAMA Pediatrics.

[45] Streissguth A (1991). Fetal alcohol syndrome in adolescents and adults. JAMA.

[46] Klingenberg, C. P. IN *Advances in morphometrics* (eds Leslie F Marcus *et al*.) 23-49 (Springer, 1996).

