# Supplementary material for: Modelling 3D craniofacial growth trajectories for population comparison and classification illustrated using sex-differences
Source: Sci Rep. 2018 Mar 19;8:4771. doi: 10.1038/s41598-018-22752-5 (PMC5859289; doi:10.1038/s41598-018-22752-5)
Supplement: Supplementary file 1 — Supplementary Information [file 41598_2018_22752_MOESM1_ESM.pdf]

Modelling 3D craniofacial growth trajectories for population comparison and classification illustrated using sex-differences

Harold S. Matthews[1,2,3,*], Anthony J. Penington[1,2,3], Rita Hardiman[4], Yi Fan[1,4], John G. Clement[1,4,5], Nicola M. Kilpatrick[1,2,3], Peter D. Claes[1,6,7]

[1] Murdoch Children's Research Institute, Melbourne, Australia
[2] Royal Children's Hospital, Melbourne, Australia
[3] Department of Paediatrics, University of Melbourne, Melbourne, Australia
[4] Melbourne Dental School, University of Melbourne, Melbourne, Australia
[5] Cranfield University, Cranfield, UK
[6] Processing Speech and Images, Department of Electrical Engineering, Katholieke Universiteit, Leuven, Belgium
[7] Medical Imaging Research Centre, Universitair Ziekenhuis, Leuven, Belgium
[*]Corresponding author
Email: harry.matthews@mcri.edu.au
Ph: +61 3 9936 6156

Supplementary Text S1

1 Fitting the Kernel Regression

1.1 Intuitive Description

Defining growth trajectories can be thought of as charting a curve through shape space. Each point on the curve corresponds to an expected head for a given age (*a*). Each point on the curve is defined by fitting a linear partial least-squares regression model, weighted so as to maximise the influence of observations closest in age to a and to minimise the influence of observations further away. This regression is used to predict the expected head and growth vectors at each point on the head at age a. The model fitting is illustrated schematically in the supplementary file Movie S1.

1.2 Weighted Partial Least-Squares Regression

Each image *f* is represented by a vector containing the co-ordinates of all *k* sampled points, after images are aligned to the sample mean and scaled to unit size by generalised Procrustes analysis.

$$f = [x_1, y_1, z_1 \dots x_k, y_k, z_k]$$

The sample of images is represented as an n (observations) x (kx3) co-ordinates matrix Y, where each row corresponds to an image. In the regression of size Y is an n x 1 matrix containing size for each observation. The matrix of predictor variables X contains only one variable (age) and is an n x 1 matrix where each row contains the age (in years) of the observation in the corresponding row of Y. Weights for each observation were calculated as described below in section 1.3. Each matrix was centred on its weighted column means ($\bar{y}$ and $\bar{x}$). Then each row of each matrix was multiplied by its corresponding weight. Partial least-squares regression of Y onto X was then performed (without further centring) using the SIMPLS algorithm, described in detail elsewhere[1]. This defines, for each variable in X, a vector of *k*x3 regression coefficients *m* that describe how each co-ordinate changes per unit of the predictor variable (per year). As there was only one predictor in the regression there is only one regression vector *m*:

$$m = \left[c_{x_1}, c_{y_1}, c_{z_1} \dots c_{x_k}, \ c_{y_k}, c_{z_k}\right]$$

where, for example c_{x_i}, represents a scalar regression coefficient describing the change in the x co-ordinate of the ith point on the image.

The expected head (*e*) at age a is produced by evaluating the regression at a and adding the mean co-ordinates $\bar{y}$ back on.

$$e = a \cdot m + \bar{y}$$

The vector *m* also defines the predicted growth vectors at each point. Specifically the growth vector at point i is:

$$[c_{x_i}, c_{y_i}, c_{z_i}]$$

This describes the predicted change in three dimensions of that point on the head.

1.3 Weighting

In the weighted partial least-squares regression each observation is assigned a weight that reflects how close its age is to a. Specifically the observation's age x_i is converted into a z-score according to a normal distribution centered on a with standard deviation σ.

$$z_i = \frac{x_i - a}{\sigma}$$

The weight for each observation w_i is then calculated by evaluating the standard normal probability density function at z_i:

$$w_i = \frac{1}{\sqrt{2\pi}} e^{\frac{-z_i^2}{2}}$$

1.4 Tuning σ

The value of σ in the above equation determines the width of the kernel. The effect of increasing σ is to use a wider range of ages to define the local linear regression. This linearises the overall kernel regression. (Figure S1.1). The value of σ must be tuned so as to best model the data. If σ is too small, then the regression will model noise in the sample rather than the true population trend (the model will be over-fitted). If σ is too large detail in the true relationship will be lost (the model will be under-fitted).

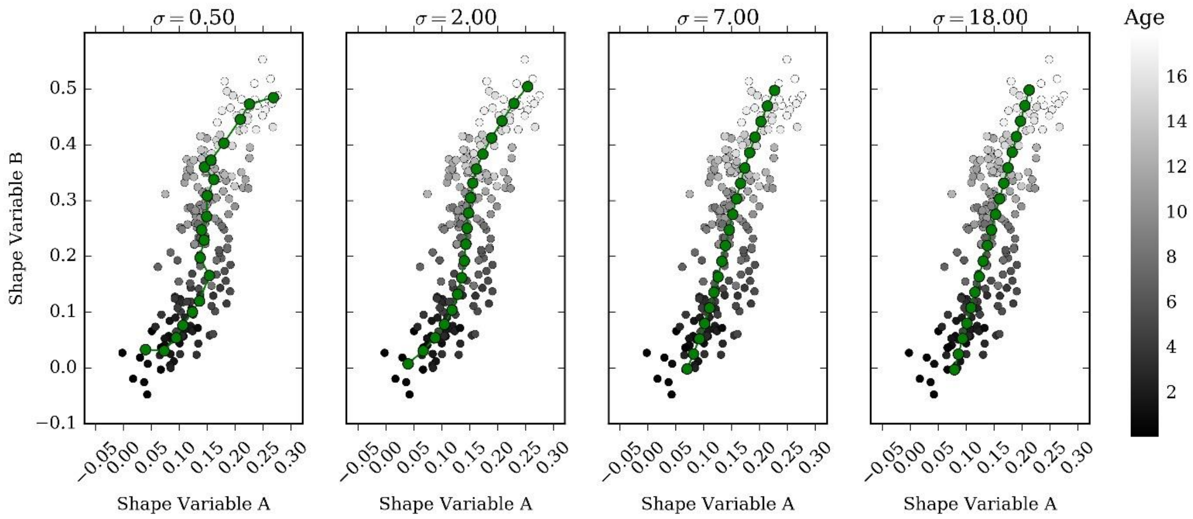

Figure Text S1.1 The effect of altering sigma on the fitted model. From left to right this shows the kernel regression model fitted to the same data using different values of σ. As σ increases the regression curve becomes more linear.

In general an over-fitted or under-fitted model will 'explain' data not used to build it (unseen data), more poorly than will a well-fitted model. In order to determine the appropriate σ we use a repeated one-dimensional grid search[2] to estimate the error on unseen data for a user-selected sample of possible values for σ.

To estimate the error for each σ the sample is split into 10 'folds'. To ensure the sampling in each fold is as even and consistent as possible these folds are constructed so that the number of observations of each age is proportional to the numbers in the total sample. For each fold the model is fitted using the other nine folds. For each observation in the test fold, error is calculated as the sum of absolute differences in each point co-ordinate between it and its age

appropriate expected head. Each fold serves as the test fold in turn and thereby an error is computed for each observation in the sample. The sum of errors over all observations is taken as the error for the value of σ. This was repeated 50 times for each σ and the mean error from thee repetitions was taken as the final error value.

We performed this separately for boys and girls and estimated an optimal σ (the one with lowest error) for each of the two samples. We then used the most conservative (largest) σ of the two to fit the models to both samples. The rationale for using the same σ for both samples is described below in section 2.3.

2 Simulations

In these simulations we use the simulated dataset shown in Figure S1.2. Here the colour of each marker represents the age. The two axes represent two response variables (e.g. axes of shape space). The data were generated by randomly sampling 200 ages between zero and eighteen. Their x and y co-ordinates were predicted from the function plotted in blue below. Random fluctuations from this true population trend were simulated by adding random Gaussian noise to the co-ordinates. The better a regression is the more closely it will estimate this true population trend from noisy data.

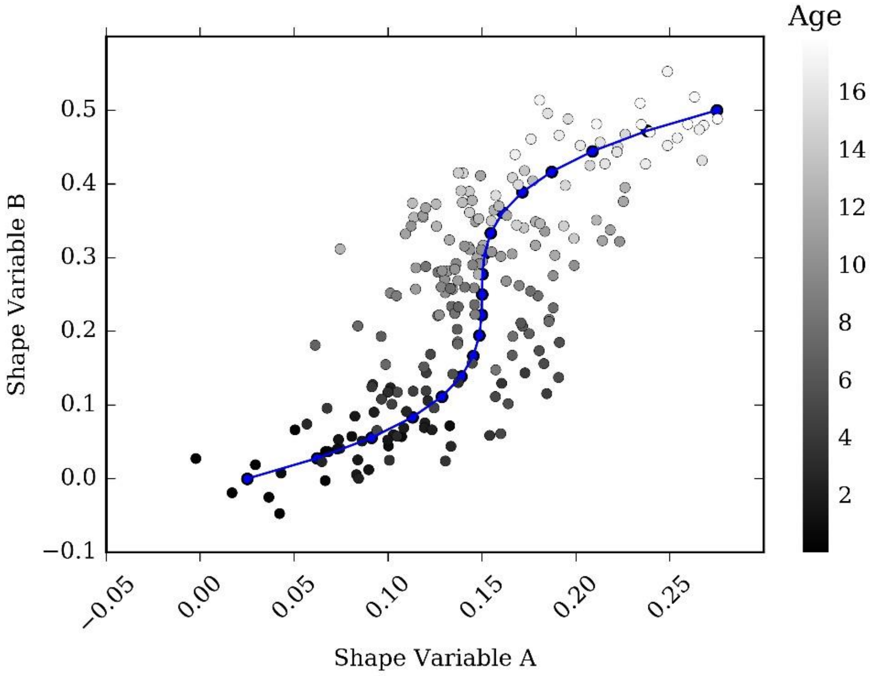

Figure Text S1.2 Simulated data and true function. The spacing of points on the true function correspond to one year intervals.

2.1 Performance under uneven sampling

Previous work has used the weighted mean head as the expected head [3]. In contrast we use a weighted partial least-squares regression function to predict the expected head. This approach is more robust when data is unevenly sampled.

Data are always unevenly sampled at the edges of the range of ages. To further illustrate the point we also removed 75% of observations from the middle of the age range. We then fitted a kernel regression as described above, with σ equal to 2.5 years. We compare this with the

weighted means calculated using the same weights as the kernel regression (see Figure S1.3). The curve of the weighted means is truncated relative to the true function at its ends. The points on this curve are also more spaced out relative to the true function where the data is more poorly sampled. Essentially the points on this curve are ‘pulled’ from areas of low sampling towards areas of higher sampling. By contrast the regression in the left panel much more closely approximates the true function.

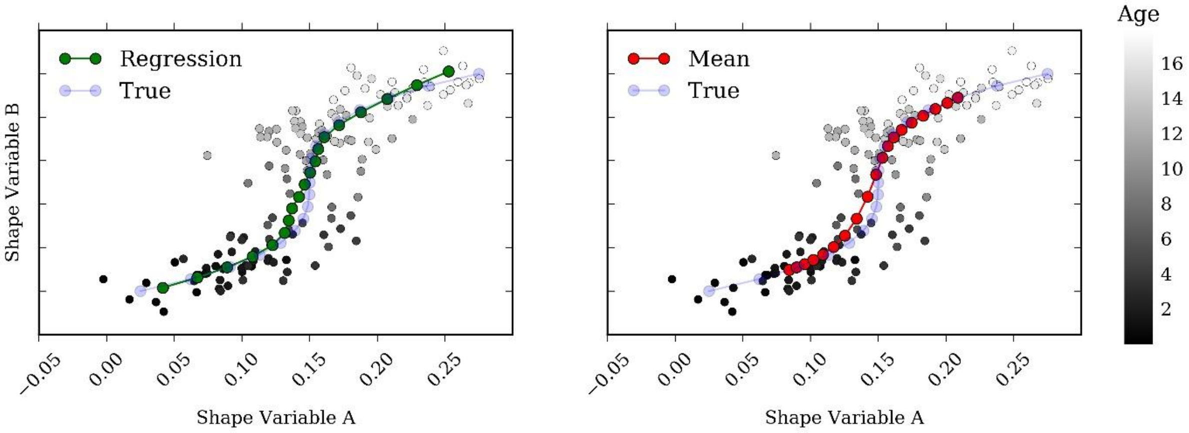

Figure Text S1.3 Compares the kernel regression fitted as described in section 1 (left) to the weighted means calculated using the same weights (right).

2.2 Performance on small sample sizes

In general, as sample size decreases a larger σ is required to avoid over-fitting. As the effect of increasing σ is to linearize the regression, this is also the effect of reducing sample size on the regression. We tuned σ and fitted the kernel regression using different sample sizes. As sample size decreases the, optimised σ becomes larger and the regression becomes more linear (Figure S1.4).

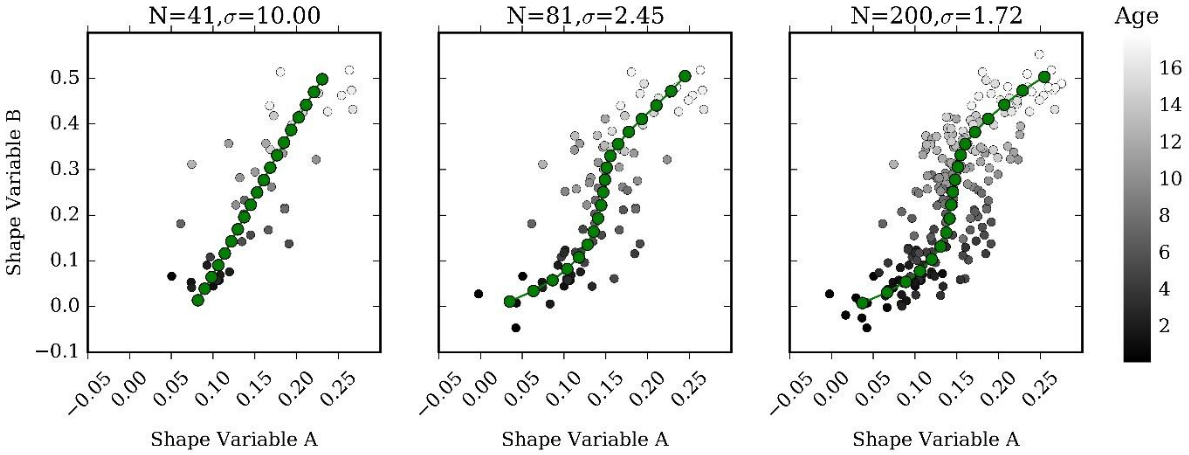

Figure Text S1.4 Regression model behaviour under small sample sizes. For each sample, σ was tuned and the regression model was fitted.

2.3 Justification for using the same σ to model both populations.

In our analysis we estimated the optimal value of σ for each sample (boys and girls) and used the most conservative (larger) to fit models to both samples. This is justified because, everything else being equal, fitting with different values of σ introduces differences in the estimated growth trajectories when they do not exist. In each panel of Figure S1.5 two

samples are plotted with identical growth trajectories (they were generated from the same true function). In the left panel the two samples are fitted with different values of σ, whereas in the right panel they are fitted using the same σ. In the left panel there are large differences introduced by one regression being more smoothed (more linear) than the other. In the right panel the two trajectories correctly appear very similar (the fact that they are not identical reflects sampling error).

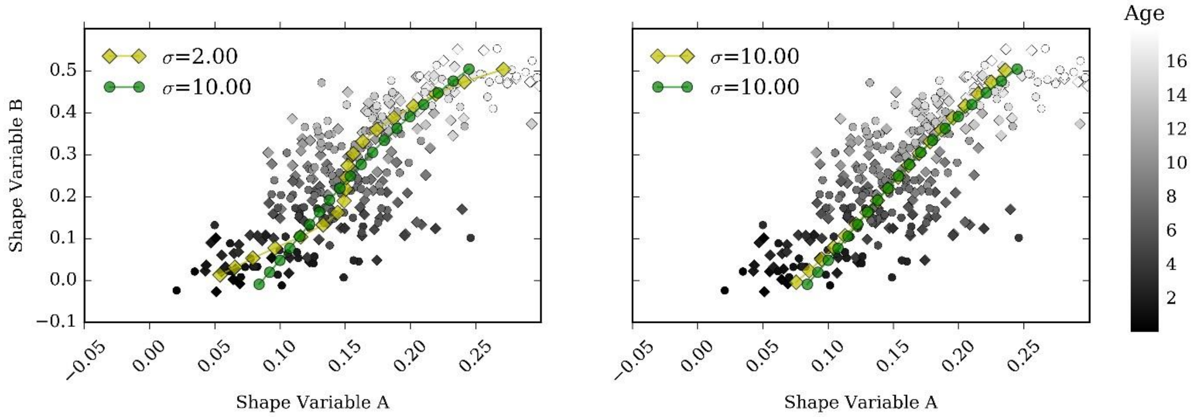

Figure Text S1.5 Compares fitting with different σ to fitting with the same σ. Both panels contain two different datasets (triangles and circles) generated using the same true function as their growth trajectory. When these are fitted with kernel regressions with different values of σ (left) spurious differences between them are introduced, whereas when they are fitted with the same σ they appear (correctly) more similar.

References

1 De Jong, S. SIMPLS: An alternative approach to partial least squares regression. *Chemometr. Intell. Lab.* **18**, 251-263, (1993).

2 Krstajic, D., Buturovic, L. J., Leahy, D. E. & Thomas, S. Cross-validation pitfalls when selecting and assessing regression and classification models. *J. Cheminform.* **6**, 10, (2014).

3 Hutton, T. J., Buxton, B. F., Hammond, P. & Potts, H. W. Estimating average growth trajectories in shape-space using kernel smoothing. *IEEE Trans. Med. Imaging* **22**, 747-753, (2003).

Table S1. Exact p-values of the Procrustes distance statistic for each age. Values less than .0001 are truncated as "<.0001".

Age	p
1.12	0.0023
1.5	0.0016
2	0.0005
2.5	0.0002
3	<0.0001
3.5	<0.0001
4	<0.0001
4.5	<0.0001
5.5	<0.0001
6	<0.0001
6.5	<0.0001
7	<0.0001
7.5	<0.0001
8	<0.0001
8.5	<0.0001
9	<0.0001
9.5	<0.0001
10	<0.0001
10.5	<0.0001
11	<0.0001
11.5	<0.0001
12	<0.0001
12.5	<0.0001
13	<0.0001
13.5	<0.0001
14	<0.0001
14.5	<0.0001
15	<0.0001
15.5	<0.0001
16	<0.0001

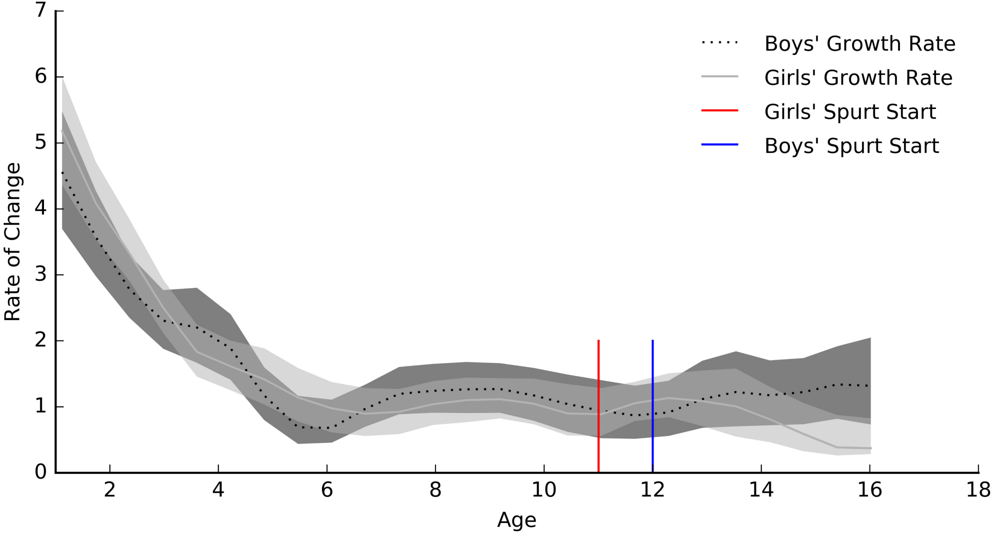

Figure S1. Comparison of male and female growth rates in size and shape together. Filled regions indicate the 95 percent confidence intervals, calculated by resampling with replacement and refitting the model 1000 times. The figure is annotated with the estimated starting points of the typical boys' and girls' growth spurts.

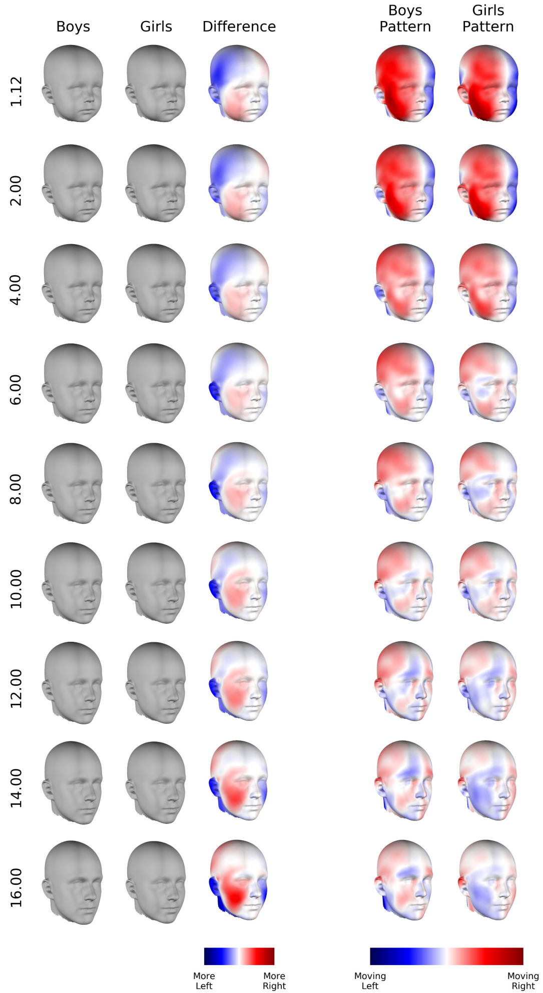

Figure S2. Growth patterns and comparison of expected images in the lateral (viewer's left/right) direction. The first three columns describe sexual dimorphism. The grey heads are the expected images. 'Shape difference' indicates how the expected images are different in the lateral direction. Blue indicates points are more to the left on the boys' images than the girls' images. Red indicates the points are more to the right. The last two columns illustrate the growth patterns of boys and girls. These indicate the amount of change occurring in the left/right direction at each point. Stronger colours indicate more change.

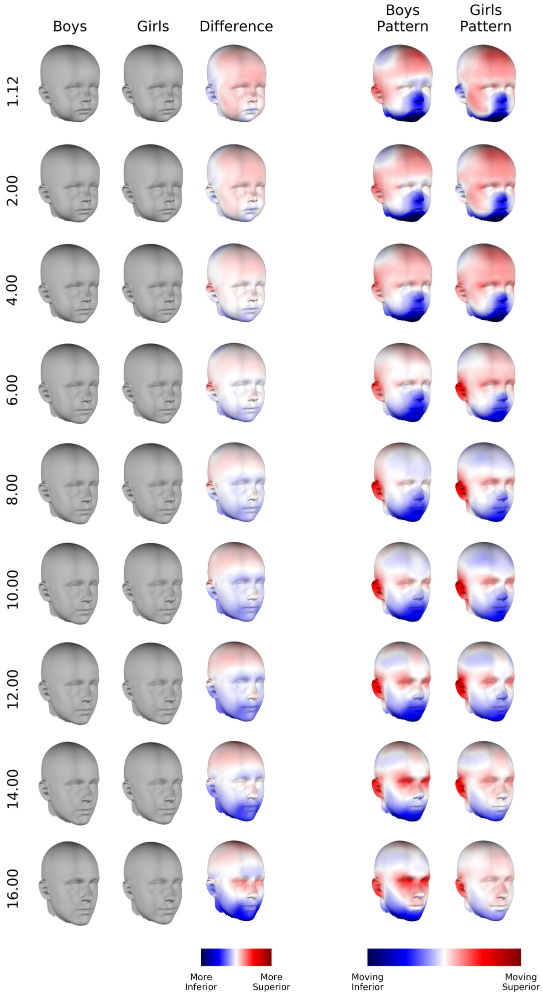

Figure S3. Growth patterns and comparison of expected images in the vertical (superior/inferior) direction. The first three columns describe sexual dimorphism. The grey heads are the expected images. 'Shape difference' indicates how the expected images are different in the vertical direction. Blue indicates points are more inferior in boys' images than the girls' images. Red indicates the points are more superior. The last two columns illustrate the growth patterns of boys and girls. These indicate the amount of change occurring in the vertical direction at each point. Stronger colours indicate more change.

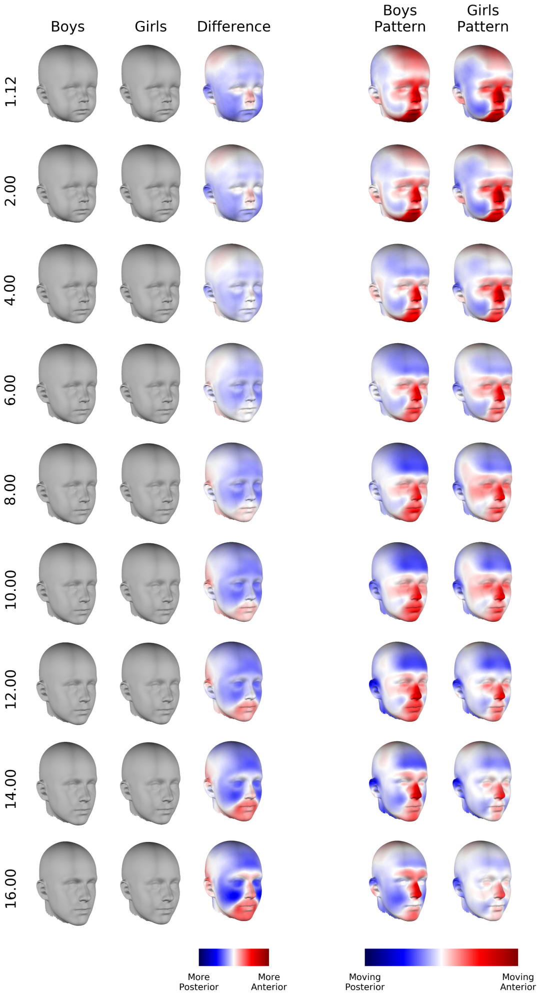

Figure S4. Growth patterns and comparison of expected images in the depth (anterior/posterior) direction. The first three columns describe sexual dimorphism. The grey heads are the expected images. 'Shape difference' indicates how the expected images are different in the depth direction. Blue indicates points are more posterior in boys' images than the girls' images. Red indicates the points are more anterior. The last two columns illustrate the growth patterns of boys and girls. These indicate the amount of change occurring in the depth direction at each point. Stronger colours indicate more change.

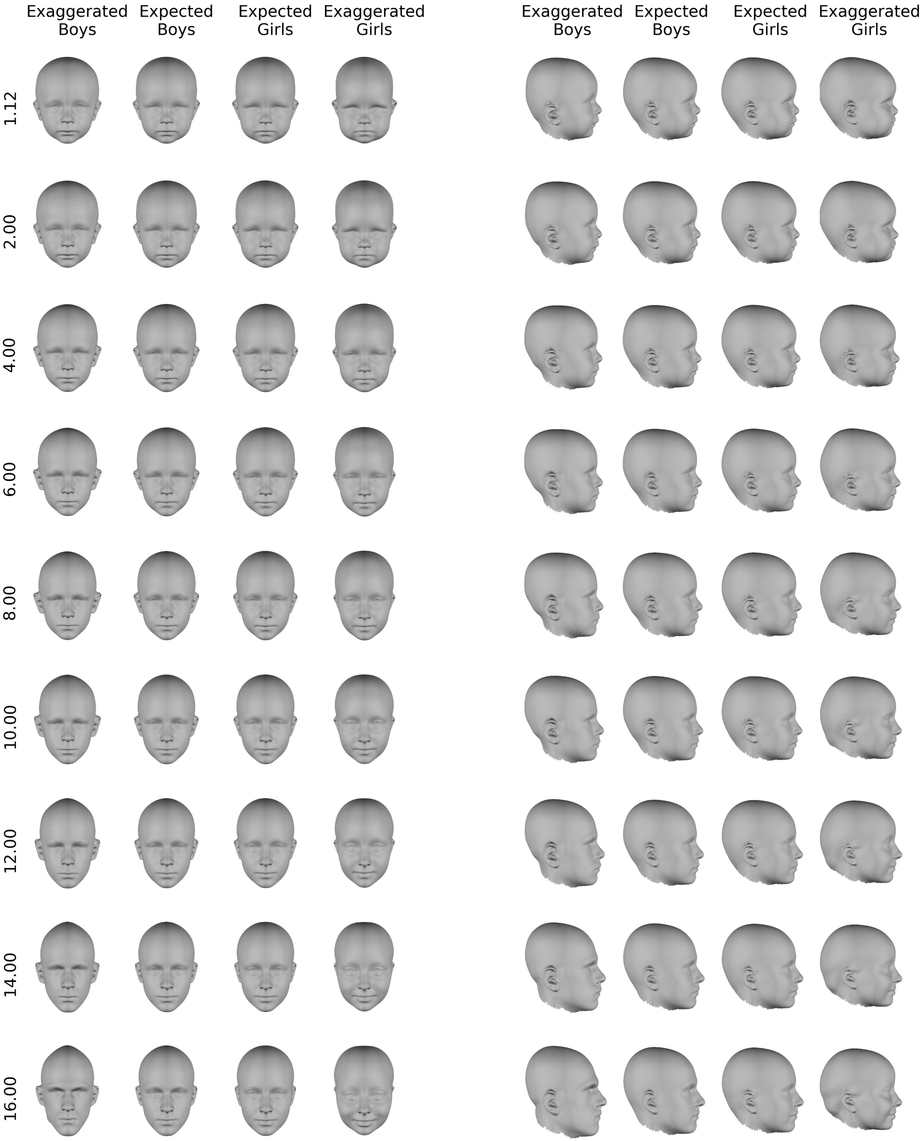

Figure S5. Expected and exaggerated boys' and girls' images. Each set of four columns contains the expected boys and girls faces in the middle two columns and the exaggerated boys and girls in the outer columns. Exaggerated faces were produced by multiplying the difference the between expected boys and girls images by two and then adding this onto the expected faces.
